# Does weight gain, throughout 15 years follow-up after Nissen laparoscopic fundoplication, compromise reflux symptoms control?

**DOI:** 10.1590/0102-672020190001e1488

**Published:** 2020-05-18

**Authors:** Victor Ramos Mussa DIB, Almino Cardoso RAMOS, Nilton Tokio KAWAHARA, Josemberg Marins CAMPOS, João Caetano MARCHESINI, Manoel GALVÃO-NETO, Adriana Gonçalves Daumas Pinheiro GUIMARÃES, Adriano Pessoa PICANÇO-JUNIOR, Carlos Eduardo DOMENE

**Affiliations:** 1 Victor Dib Institute, Manaus, AM, Brasil; 2 Gastro Obeso Center, São Paulo, SP, Brasil; 3 School of Medicine, São Paulo University, São Paulo, SP, Brazil; 4 Federal University of Pernambuco, Recife, PE, Brazil; 5 Caetano Marchesini Clinic, Curitiba, PR, Brazil; 6 Airforce Surgical Center, Manaus, AM, Brazil; 7 Federal University of Amazonas, Manaus, AM, Brazil

**Keywords:** Gastroesophageal reflux, Fundoplication, Endoscopy, Refluxo gastroesofágico, Fundoplicatura, Endoscopia

## Abstract

***Background*:**

Gastroesophageal reflux disease is defined by the abnormal presence of gastric content in the esophagus, with 10% incidence in the Western population, being fundoplication one treatment option.

***Aim*:**

To evaluate the early (six months) and late (15 years) effectiveness of laparoscopic fundoplication, the long term postoperative weight changes, as well as the impact of weight gain in symptoms control.

***Methods*:**

Prospective study of 40 subjects who underwent laparoscopic Nissen’s fundoplication. Preoperatively and early postoperatively, clinical, endoscopic, radiologic, manometric and pHmetric evaluations were carried out. After 15 years, clinical and endoscopic assessments were carried out and the results compared with the early ones. The presence or absence of obesity was stratified in both early and late phases, and its influence in the long-term results of fundoplication was studied, measuring quality of life according to the Visick criteria.

***Results*:**

The mean preoperative ages, weight, and body mass index were respectively, 51 years, 69.67 kg and 25.68 kg/m^2^. The intraoperative and postoperative complications rates were 12.5% and 15%, without mortality. In the early postoperative period the symptoms were well controlled, hernias and esophagitis disappeared, the lower esophageal sphincter had functional improvement, and pHmetry parameters normalized. In the late follow-up 29 subjects were assessed. During this period there was adequate clinical control of reflux regardless of weight gain. In both time periods Visick criteria improved.

***Conclusion*:**

Fundoplication was safe and effective in early and late periods. There was late weight gain, which did not influence effective symptoms control.

## RESUMO

***Racional*:**

A doença do refluxo gastroesofágico é definida pela presença anormal do conteúdo gástrico no esôfago com incidência de 10% na população ocidental, sendo a fundoplicatura uma das opções de tratamento.

***Objetivo*:**

Avaliar a efetividade precoce (seis meses) e tardia (15 anos) da fundoplicatura laparoscópica, bem como a evolução ponderal pós-operatória em longo prazo, e o impacto do ganho de peso no controle tardio dos sintomas.

***Métodos*:**

Estudo prospectivo com 40 indivíduos submetidos à fundoplicatura laparoscópica pela técnica de Nissen. No pré e pós-operatório precoce, foram realizadas avaliações clínica, endoscópica, radiológica, manométrica e pHmétrica. Após 15 anos, realizaram-se avaliações clínica e endoscópica, comparando-se os resultados com os da fase precoce. Estratificou-se a presença ou ausência de obesidade nestas fases e estudou-se sua influência nos resultados em longo prazo, mensurando-se a qualidade de vida pelos critérios de Visick.

As médias de idade, peso e do índice de massa corporal pré-operatórias foram 51 anos, 69.67 kg e 25,68 kg/m^2^. O índice de complicações intra e pós-operatórias foram 12,5% e 15%, sem mortalidade. No pós-operatório precoce houve controle dos sintomas, remissão das hérnias e esofagites, melhora funcional do esfíncter esofágico inferior e normalização dos parâmetros pHmétricos. No seguimento tardio, 29 indivíduos foram acompanhados. Nesta fase, houve adequado controle clínico do refluxo, independente do ganho de peso. Em ambas as fases houve melhora nos critérios de Visick.

***Conclus*ão:**

A fundoplicatura foi segura e efetiva, precoce e tardiamente. Houve ganho de peso tardio, o que não influenciou no controle efetivo dos sintomas.

## INTRODUCTION

Gastroesophageal reflux disease (GERD) is defined by the abnormal presence of gastric content above the lower esophageal sphincter (LES), causing a variable spectrum of symptoms and complications^30^. It affects approximately 10% of the Western population^18^ ^,^ ^19^. The most frequent clinical manifestations are pyrosis and regurgitation^29^. The disease is often controlled through clinical measures^12^ and surgery is reserved for patients with failed clinical treatment, advanced erosive esophagitis, Barrett’s esophagus, peptic stenosis or when the patient demands the operation^12^ ^,^ ^20^ ^,^ ^28^ ^,^ ^29^ ^,^ ^34^. Despite the clinical treatment, some risk factors for disease progression are nocturnal reflux, low-pressure LES, and early erosive esophagitis^9^.

Minimally invasive surgery has rendered fundoplication more frequent^9^ with results similar to laparotomy^3^ ^,^ ^30^. However, the long-term surgical effectiveness is still a matter of discussion^30^.

Postoperative weight gain may lead to fundoplication failure to contain reflux due to the increase in intra-abdominal pressure and modification of the gastroesophageal pressure gradient^14^ ^,^ ^23^. In this case, a new surgery may be necessary to control weight and preserve the anti-reflux mechanism. Gastric bypass with jejunal diversion seems to be the best option for this purpose, however with relevant associated morbidity^29^.

The objectives of this study were to evaluate the safety of laparoscopic fundoplication, its effectiveness in the early (six months) and late (15 years) control of reflux symptoms, and the influence of postoperative weight gain on late reflux control. 

## METHODS

Forty subjects with GERD-compatible symptoms (pyrosis, regurgitation, dysphagia), diagnosed by clinical investigation and complementary exams, who had failed clinical treatment, were prospectively evaluated. They underwent total laparoscopic fundoplication (Nissen) between March and September 2001.

 The study was approved by the local ethics and research committee of the Prontocord Hospital in Manaus, AM, Brazil, and all patients agreed to participate by signing the informed consent form.

The inclusion criteria were age between 18-70 years and disease durationfor a minimum of six months. Patients with severe pulmonary or heart disease, coagulopathies, previous gastroesophageal surgery, esophageal aperistalsis, and morbid obesity were excluded.

Upper digestive endoscopy, contrast esophageal radiogram, esophageal manometry and prolonged esophageal pH monitoring, were done for preoperative evaluation of gastroesophageal reflux. The surgical indication was based on reflux symptoms and, at least, two altered complementary evaluations, regarding the following findings: hiatal hernia and/or any level of erosive esophagitis, according to the modified Savary-Miller classification; hypotonic LES; pathological reflux and/or physiological reflux with positive symptom index.

Nissen fundoplication was done by laparoscopy in all cases. Venous and inhalation general anesthesia was used, positioning the patient in horizontal supine position and with the lower limbs slightly open, the surgeon standing between them. The abdominal cavity was accessed after CO_2_ pneumoperitoneum was done, inserting five portals arranged as follows: below the xiphoid appendix (5 mm), right hypochondrium (5 mm), left flank (5 mm), left hypochondrium (10 mm) and supraumbilical (10 mm).

The diaphragmatic crura were approximated using two or three separate Ethibond 2-0 sutures, calibrated by a 36F bougie. The proximal short vessels were dissected and a 360^o^ valve was constructed with the gastric fundus that circled the distal esophagus for 3 cm, shaped by a 36F Fouchet (floppy) catheter. Three or four separate Ethibond 2-0 sutures were used. All procedures, as well as pre- and postoperative evaluations were carried out by the same surgeon and team.

The surgical performance was evaluated through operative time, surgical complications and conversions rates. The efficacy of the procedure in correcting reflux was assessed at six months, on average, by clinical investigation of reflux symptoms and by the need for proton pump inhibitors (PPIs), also applying the Visick score, as follows: grade I - absence of gastrointestinal symptoms after the operation; grade II - mild symptoms, such as discomfort after meals, food intolerance, malaise, controlled with rest and dietary restriction; grade III - uncontrollable symptoms with general care, such as diarrhea, vomiting or epigastric pain, requiring medications; grade IV - symptoms equal to or worse than those prior to the operation, disease relapse or complication.

In the early postoperative period (six months), the previously mentioned complementary exams were conducted to compare with the results obtained preoperatively. There was subsequent 15-year follow-up, observing the subjects’ weight progression and the possible impact of weight gain on the control of reflux symptoms. In case of morbid obesity, with or without recurrence of reflux, videolaparoscopic bariatric surgeries would be proposed and those patients excluded from the study. Late clinical evaluation of the sample, after 15 years, was carried out through clinical inquiry on the recurrence or not of reflux symptoms and the need or not of PPIs, using also the Visick score. Upper digestive endoscopy was also done to analyze the valve, alterations of the esophageal hiatus and the presence or absence of erosive esophagitis. These evaluations were stratified according to present or absent obesity. The patients were searched through telephone contact and e-mail.

### Statistical analysis

The SAS (System Analysis Statistical) software was used for statistical analysis. In the quantitative data, when the hypothesis of normality was accepted, the Shapiro-Wilk test was used to calculate mean, standard deviation, and the Student t-test was used for paired data. When the hypothesis of normality was rejected, the median was calculated and the non-parametric Wilcoxon test applied. In the analysis of categorical variables, the chi-square test was applied or when that was not possible, the Fisher’s exact test. A significance level of 5% was set for the application of statistical tests.

## RESULTS

Forty individuals were preoperatively evaluated in this study, 29 of them females (72.5%). The average preoperative GERD duration was 76 months; the average age 51 years, and the average BMI 25.68 kg/m^2^.

### Preoperative and early postoperative clinical results

In the preoperative period 90% of the subjects complained of moderate pyrosis, 47.5% regurgitation and 55% dysphagia. At six months postoperatively, complaints of both pyrosis and regurgitation decreased to 2.5%, and dysphagia to 7.5%. Using the Visick criteria at six months, 77.5% of the subjects were classified as grade I; 15% as grade II; and 7.5% as grade III.

### Preoperative and early postoperative results of complementary exams

Endoscopy was used to evaluate hiatal hernia and esophagitis. Comparing preoperative and early postoperative data, a reduction from 97.5% to 10% (p<0.001) was found in the percentage of hernias, detecting one case of paraesophageal hernia after fundoplication. There was a reduction from 77.5% to 7.5% in esophagitis, only the cases of Barrett’s esophagus remained. In postoperative follow-up esophagogram, there was a significant reduction in hiatal hernias and manometry showed an increase in the percentage of subjects with normal LES (Table 1). 

**TABLE 1:** Distribution according to the preoperative and early postoperative results of complementary exams

Variables	Assessment				
	Pre		Post		
	f_i_	%	f_i_	%	p*
Endoscopy- axial hiatal hernia					<0.001
Yes	39	97.5	4	10.0	
No	1	2.5	36	90.0	
Total	40	100.0	40	100.0	
Esophagitis					<0.001
Yes	30	75.0	3	7.5	
No	10	25.0	37	92.5	
Total	40	100.0	40	100.0	
Esophagogram- hiatal hernia					<0.001
Yes	26	89.7	6	15.0	
No	3	10.3	34	85.0	
Total	29	100.0	40	100.0	
Manometry (LES tonus)					<0.001
hypotonic LES	27	67.5	7	18.4	
normal LES	13	32.5	31	81.6	
Total	40	100.0	38	100.0	

f_i_=absolute simple frequency; *p value in bold indicates statistical difference between proportions at 5% (p<0.05), using exact Fisher’s test

There was significant (p<0.001) postoperative improvement in the esophageal manometry that assessed the LES tonus and its total and abdominal lengths. Significant postoperative reflux control was demonstrated in pHmetry (Table 2).

**TABLE 2:** Comparison according to pre and early (six months) postoperative complementary exams

Variables	Assessment		p*
Manometry	Pre	Post	
Total median LES length (cm)	3.6	4.8	<0.001
Abdominal median LES length (cm)	1.2	3.0	<0.001
LES median pressure (mmHg)	6.6	19.2	<0.001
pHmetry			
Total time % median pH <4	7.5	0.3	<0.001
Orthostatic % median pH <4	6.8	0.9	<0.001
Supine % median pH <4	6.8	0.4	<0.001

*p value in bold indicates statistical difference between proportions at 5% (p<0.05), using exact Wilcoxon’s test

### Surgical technique-related results

Laparoscopy Nissen fundoplication was done in 40 subjects. Intraoperative and postoperative complications rates were 12.5% and 15%, respectively (Table 3). There was one case of liver trauma from the liver retractor, causing liver bleeding and leading to conversion to laparotomy, for bleeding control. The average surgical time was 97.4 min and the hospital length of stay, 17.9 h. Feeding was started on average 9 h after the procedure.

**TABLE 3:** Frequency of trans and postoperative complications of fundoplication

Trans operative complications	n	%
Large cervical emphysema Short vessels bleeding Liver bleeding Left inferior phrenic vein injury	01 01 02 01	2.5 2.5 5 2.5
Total	05	12.5
Postoperative complications (within 30 days)		
Persistent vomiting on 1^st^ POD Adynamic ileus until 2^nd^ POD Chest pain Intense hiccups on 1^st^ POD Paraesophageal hernia Operative wound infection	01 01 01 01 01 01	2.5 2.5 2.5 2.5 2.5 2.5
Total	06	15

POD=postoperative day

### Late follow-up

After 15 years of fundoplication, clinical, endoscopic and weight aspects of 29 subjects were evaluated (72.5% of the initial sample). It was not possible to evaluate 11 patients from the initial sample (two of them were excluded from the study after undergoing bariatric techniques, to treat obesity).

Regarding the weight change of the sample, there was a mean increase in BMI of 3.64 kg/m^2^ in the final evaluation (p<0.001), with significant development of obesity (Table 4).

Regarding the symptoms of pyrosis and regurgitation, there was a significant reduction in the late postoperative period, as compared to the preoperative (Table 4). Subjects who reported late pyrosis mentioned lower intensity and frequency, as compared to the preoperative period. There were other sporadic complaints that included dysphagia, non-cardiac chest pain, and globus*.*

**TABLE 4:** Comparison of pre and late postoperative BMI and symptoms

Variables	Preoperative assessment (n=40)		15-year follow up (n=29)		P
	f_i_	%	f_i_	%	
BMI (kg/m^2^)					<0.001*
Normal weight (20-24.9)	22	55.0	01	3.4	
Overweight (25-29.9)	17	42.5	16	55.2	
Obesity (30-34.9)	1	2.5	12	41.4	
Mean BMI±SD	25.68 ± 1.54		29.32 ± 2.53		
Symptoms					
None	-	-	15	51.7	<0.001**
Pyrosis	30	90.0	10	34.5	<0.001**
Regurgitation	19	47.5	-	-	<0.001**
NCCP***	2	5.0	2	6.9	0.999**
Dysphagia	10	25.0	3	10.3	0.218**
Globus	1	2.5	1	3.4	0.999**

f_i_=absolute simple frequency; SD=standard deviation; NCCP=non-cardiac chest pain; *p value in bold indicates statistical difference at 5% (p<0.05), in relation to the averages, using Sudent’s *t* test;***=exact Fisher’s test

Table 5 depicts the comparison between preoperative and late (15-year) endoscopic findings, demonstrating consistent persistence of surgical anatomy and effective control of erosive esophagitis.

There was one case of late esophagitis on a Barrett’s patient, who persisted after fundoplication, with no histopathological dysplasia. It is important to note that two other cases of Barrett found in the preoperative period were among the subjects not evaluated at this stage.

**TABLE 5:** Comparison of pre and late (15 years) endoscopic findings

Variables	Assessment				
	Pre		Late post		
	f_i_	%	f_i_	%	p*
Esophagitis					<0.001
Yes	30	75.0	3	10.3	
No	10	25.0	26	89.7	
Total	40	100.0	29	100.0	
Axial hiatal hernia					<0.001
Yes	39	97.5	2	6.9	
No	1	2.5	27	93.1	
Total	40	100.0	29	100.0	

f_i_=absolute simple frequency; *p value in bold indicates statistical difference between proportions at 5% (p<0.05), using exact Fisher’s test

Table 6 depicts the late comparative assessment between the patients who developed obesity and those who did not, considering clinical and endoscopic findings, the level of satisfaction with the surgical results according to the Visick scale, as well as the need to use PPIs. On such assessments no statistical difference was found between the two groups. 

**TABLE 6:** Comparison of clinical and endoscopic variables regarding late (15 years) postoperative weight

Variables	Obesity				Total	p*
	Yes (n=12)		No (n=17)			
	fi	%	fi	%		
Esophagitis						0.998
Yes	1	8.3	2	11.8	3	
No	11	91.7	15	88.2	26	
Hiatal hernia						0.999
No	11	91.7	16	94.1	27	
Yes	1	8.3	1	5.9	2	
Use of PPIs						0.999
Yes	6	50.0	8	47.1	14	
No	6	50.0	9	52.9	15	
Visick classification						0.919
I	6	50.0	9	52.9	15	
II	5	41.7	6	35.3	11	
III	1	8.3	2	11.8	3	
Symptoms						0.999
None	6	50,0	9	52,9	15	
Pyrosis	5	41,7	5	29,5	10	
NCCP	1	8,3	1	5,9	2	
Dysphagia	1	8,3	2	11,8	3	
Globus	1	8,3	-	-	1	

f_i_=absolute simple frequency; * =exact Fisher’s test, NCCP=non-cardiac chest pain

## DISCUSSION

GERD is usually approached through clinical treatment, intending to control reflux symptoms and heal esophagitis. Surgical treatment, on the other hand, has the additional objectives of restoring the anatomy and function of the gastroesophageal transition^21^.

However, some uncertainties remain regarding the surgical procedure in respect to the durability of its results and to its side effects^28^. There are, therefore, still controversies regarding the indication of fundoplication for the treatment of GERD.^28^ ^,^ ^30^ According to Holscher *et al.* ^12^ surgical intervention would take place when adequate clinical control of reflux was not obtained, a parameter used in the current study, consonant to other authors^1^ ^,^ ^6^.

The introduction of the minimally invasive method for correction of gastroesophageal reflux has optimized its surgical indication^8^ ^,^ ^21^. Intraoperative complication rates range from 2-13% and conversion rates from 1-10%^5^. The complication rates of this study are consistent with the literature, with zero mortality.

There is controversy as to the type of valve to be used for the correction of reflux, and they may totally or partially encircle the gastroesophageal transition^4^ ^,^ ^18^ ^,^ ^31^ ^,^ ^32^. In a systematic review Ramos *et al*. ^25^ concluded that partial fundoplication has fewer obstructive side effects as compared to total. In contrast, supporters of the total fundoplication (Nissen) state that this type of valve is more effective in containing reflux^5^ ^,^ ^21^. In the current study, only total fundoplication was used.

Ciovica *et al.* ^7^ evaluated 550 patients who underwent laparoscopic fundoplication with a one-year postoperative follow-up. They found improvement in GERD symptoms and quality of life, only 3.2% of cases requiring antiacids. Moore *et al.* ^18^ demonstrated that surgical treatment of GERD has good reflux control, with improvement of symptoms in 85-93% of cases, favorably impacting quality of life. In the current sample, in early postoperative evaluation there was significant improvement in complaints of pyrosis and regurgitation using the Visick’s criteria^5^ ^,^ ^27^ ^,^ ^32^ to grade improvement in reflux symptoms.

In the early postoperative period of this study there was significant reduction in hiatal hernias and remission of esophagitis; however, consonant to the literature^17^ ^,^ ^33^, the cases of Barrett’s esophagus persisted and do not seem to regress after surgery, deserving systematic follow-up.

Short-term postoperative pHmetry indicated physiological reflux in all cases, demonstrating effective reflux control, corroborating some authors^11^ ^,^ ^16^. At esophageal manometry, significant LES restoration was noted regarding its length and pressure, indicating the importance of the operation in restoring the functionality of the gastroesophageal transition28.

The esophagogram was conducted in all cases in the early postoperative period in order to evaluate the anatomy of the gastroesophageal transitional region and to verify the position and extension of the valve. Paraesophageal hiatal hernia was detected in one case. Awad *et al.* ^2^ reinforce the importance of barium esophagogram in the diagnosis and routine postoperative follow-up of these hernias.

Some authors document the relief of typical symptoms (pyrosis and regurgitation) at prolonged follow-up after surgical correction of GERD^11^ and demonstrate the superiority of the operation over clinical treatment^7^. Specheler *et al* ^28^ stated, however, that in the long term the operation is not so effective in containing reflux, and a substantial number of patients will need antiacids. According to Lundell *et al* ^16^ *.* this condition may indicate increased esophageal sensitivity to reflux without necessarily being pathological, which can be assessed by pHmetry. In the current sample, it was noted later that 62.5% of symptomatic subjects had no endoscopic evidence of reflux, which could reflect increased esophageal sensitivity. This cannot be confirmed, however, given that pHmetry was not employed at this stage of the evaluation, which is a limitation of this study.

In the last decades weight gain has been a worldwide trend^23^, a fact also seen in subjects submitted to fundoplication^14^. Some authors reported that weight gain is a predictive factor of recurrence of reflux symptoms after fundoplication, either due to increased intra-abdominal pressure, valve migration or rupture^1^ ^,^ ^14^ ^,^ ^22^, what is not corroborated by other authors^10^ or by this sample. In this study, after subjects stratification according to present or absent development of late obesity, there was no significant correlation between weight gain and relapse of reflux symptomatology or anatomical alteration of fundoplication after clinical and endoscopic evaluation. The use of PPIs was on demand, equal in the obese and non-obese, in a restricted portion of the sample, consonant with some studies^7^ ^,^ ^11^, and dissonant from others^6^. The use of the Visick criteria^27^ in the late phase of this evaluation, demonstrated that good results were sustained, even in subjects who developed obesity. 

The weight gain that follows the antireflux operation is usually late, and the average time for the development of obesity is six years, with possible indication of bariatric surgery^14^, consistent with the findings of this study. Conversion to bariatric technique may be indicated by reflux recurrence, obesity, or both^15^ ^,^ ^22^, and should be carried out by an experienced surgical team.^29^. In this series, 41.4% of the subjects who were followed long term, developed grade I obesity (p<0.0001).

The small sample size of this series, as well as the absence of a control group, represents limitations of this study, precluding conclusive inferences regarding results, and other studies are recommended to confirm or not these findings.

## CONCLUSION

Laparoscopic fundoplication proved to be a safe procedure, resulting in long lasting efficient control of gastroesophageal reflux. Weight gain in late follow-up did not hinder reflux symptoms control or the anatomic preservation of the valve. 
